# Serum complement C3 levels are associated with nonalcoholic fatty liver disease independently of metabolic features in Chinese population

**DOI:** 10.1038/srep23279

**Published:** 2016-03-31

**Authors:** Chengfu Xu, Yi Chen, Lei Xu, Min Miao, Youming Li, Chaohui Yu

**Affiliations:** 1 Department of Gastroenterology, the First Affiliated Hospital, College of Medicine, Zhejiang University, Hangzhou, China; 2 Department of Gastroenterology, Ningbo First Hospital, Ningbo, China; 3 Department of Internal Medicine, Zhenhai Lianhua Hospital, Ningbo, China

## Abstract

Serum complement C3 levels are closely associated with obesity and related metabolic disorders. This study aimed to investigate the association between serum complement C3 levels with non-alcoholic fatty liver disease (NAFLD). A cross-sectional study was performed among adults who took their annual health examinations at Zhenhai Lianhua Hospital, Ningbo, China during 2014. We included 7540 participants (5069 men and 2471 women) in this study. NAFLD patients had higher serum complement C3 levels (*P* < 0.001), and these levels were positively associated with both NAFLD prevalence and severity (*P* < 0.001). The above association remains true among lean and metabolic syndrome-free participants. Multivariable regression analysis showed that serum complement C3 was independently associated with risk for NAFLD (OR = 5.231; 95% CI: 3.169–8.635). Serum complement C3 level is positively associated with prevalence and severity of NAFLD, and this association is independent of obesity and metabolic syndrome.

Nonalcoholic fatty liver disease (NAFLD) is one of the most epidemic chronic liver diseases worldwide and emerging as a major public health problem globally in recent years. The prevalence of NAFLD is approximately 20% in general population and up to about 70% in patients with type 2 diabetes^1^. NAFLD represents a spectrum of liver diseases, including simple steatosis, nonalcoholic steatohepatitis (NASH), fibrosis and cirrhosis^2^. Simple steatosis is considered to be benign with slow exacerbation over decades, whereas NASH can progress to cirrhosis, liver failure and even hepatocellular carcinoma in a relatively short period of time^3^.

Obesity is a major risk factor of NAFLD and increase in body weight is a main drive for high prevalence of NAFLD^4^,^5^,^6^. Weight reduction is proved to be beneficial in NAFLD treatment, with improvement in serum liver enzyme and liver histopathology^7^. Recent studies reported that NAFLD is also commonly detected in non-obese individuals^8^,^9^,^10^, suggesting that, besides obesity, other factors may contribute to development of NAFLD.

Complement system is increasingly recognized to be closely associated with obesity and related metabolic disorders^11^, and may be involved in NAFLD. In an observational study conducted in Netherland, activation of complement system was observed in 74% of 43 NAFLD patients^12^. In another cohort of 523 subjects with (an increased risk of) type 2 diabetes and cardiovascular diseases, serum complement C3a levels were observed to be positively associated with liver fat content^13^. A recent relatively large population study in China also found that participants with higher serum complement C3 levels are more likely to have NAFLD than those with low C3 levels^14^. However, whether the association between serum complement C3 levels or NAFLD is independent of obesity and metabolic syndrome remains not certain, neither is the link between serum C3 levels and severity of NAFLD.

Herein, we performed a cross-sectional study to investigate the association of serum C3 levels with prevalence and severity of NAFLD in a large Chinese population.

## Methods

### Study population

This study was performed among adults who took their annual health examinations at Zhenhai Lianhua Hospital, Ningbo, China during 2014. We excluded the following participants: (1) those with alcohol consumption greater than 140 g/week for men and 70 g/week for women; (2) those with history of viral hepatitis, autoimmune hepatitis, or other forms of chronic liver disease; (3) those with self-reported acute infection within 2 weeks; and (4) those with body mass index less than 18.0 kg/m^2^. A total of 7540 participants (5069 men and 2471 women) were enrolled in this study. The study protocol was approved by the Hospital Ethics Committee and performed in accordance with Declaration of Helsinki. All persons gave their informed consent prior to their inclusion in the study.

### Clinical evaluations

Clinical evaluations were performed according to procedures as previously described^9^,^15^. In brief, demographic data, medical history, and health habits were recorded by trained physicians. Standing height and body weight without shoes and with light clothes were measured in standard procedures. Body mass index was calculated as body weight (kg) divided by square of height (meters). Waist circumference was measured at the level of the narrowest point between the iliac crest and the rib cage using a non-stretchable tape. Systolic and diastolic blood pressures were measured using an automated sphygmomanometer, with participants in sitting position.

Overnight fasting blood samples were obtained from each participant, and serum samples were separated for biochemical analysis without freezing. The biochemical values, including liver enzymes, serum lipids, glucose, and uric acid, were measured by a Hitachi 7600 autoanalyzer (Hitachi, Tokyo, Japan) using standard protocols. Serum complement C3 levels were assessed using immune-turbidimetric assay by a Hitachi 7600 autoanalyzer (Hitachi) using standard methods. The coefficients of variation were 4.5% and 6.6% for inter-assay and intra-assay, respectively.

### Definition of NAFLD and metabolic syndrome

NAFLD was determined by hepatic ultrasound examination following exclusion of excessive alcohol consumption, viral, or autoimmune liver disease. Hepatic ultrasound examinations were carried out by trained ultrasonographists using a Toshiba Nemio 20 sonography machine with a 3.5-MHz probe (Toshiba, Tokyo, Japan). The ultrasonographists were blinded to the study design and clinical data. The criteria for ultrasonic diagnosis of fatty liver were based on those recommended by the Chinese Liver Disease Association^16^.

Metabolic syndrome was defined as participant who has three or more abnormality of the following criteria recommended by new International Diabetes Federation^17^: (1) central obesity, defined as waist circumference ≥90 cm for Chinese men and ≥80 cm for Chinese women; (2) raised triglyceride level, defined as triglycerides ≥1.70 mmol/L or specific treatment for this lipid abnormality; (3) reduced HDL-C level, defined as HDL-C <1.03 mmol/L for men and <1.29 mmol/L for women; (4) raised blood pressure, SBP ≥130 mmHg or DBP ≥85 mmHg, or treatment of previously diagnosed hypertension; and (5) raised FPG, defined as FPG ≥5.60 mmol/L, or previously diagnosed type 2 diabetes.

### Statistics analysis

We performed statistical analyses using SPSS 18.0 software for Windows (SPSS Inc., Chicago, IL). We expressed continuous variables as mean and standard deviation or median and interquartile range, and compared through using Student’s *t*-test or Mann-Whitney *U* test. We compared categorical variables using chi-square test. We applied a multiple stepwise regression analysis (backward: Wald; cutoff for entry: 0.05, for removal: 0.10) to assess the risk factors for NAFLD. We considered a 2-tailed *P* value less than 0.05 to be statistically significant.

## Results

### Serum complement C3 levels are elevated in participants with NAFLD

Of 7540 participants enrolled in this study, 2070 (27.45%) had NAFLD. The clinical characteristics of participants with or without NAFLD were presented in Table 1. We found that participants with NAFLD were older, male predominant, and with higher body mass index, waist circumference, systolic and diastolic blood pressure than those without NAFLD (Table 1). We also found participants with NAFLD had significantly higher serum levels of alanine aminotransferase (ALT), aspartate aminotransferase (AST), γ-glutamyltransferase, albumin, triglyceride, total cholesterol, LDL cholesterol, fasting blood sugar, uric acid, white blood cell, and platelet count, while lower serum HDL cholesterol levels than those without NAFLD (all with *P* < 0.001; Table 1). A noticeable finding is that serum complement C3 levels were significantly higher in participants with NAFLD than those without NAFLD, suggesting a potential link between serum complement C3 levels with NAFLD (Table 1).

### Serum complement C3 levels are positively associated with prevalence and severity of NAFLD

To further clarify the association of serum complement C3 levels with NAFLD, we divided all participants into quartiles according to their serum complement C3 levels: <1.07 g/L, 1.07–1.16 g/L, 1.17–1.26 g/L, and ≥1.27 g/L for quartile 1, 2, 3, and 4, respectively. We found a positive correlation between serum complement C3 quartiles and NAFLD prevalence. The prevalence was 7.8% among participants with serum complement C3 in the first quartile, and increased to 19.45%, 31.56%, and 49.41%, in quartiles 2, 3, and 4, respectively (*P* for trend <0.001; Table 2). This finding suggests that participants with higher serum complement C3 quartiles are more likely to have NAFLD.

We also analyzed the association between serum complement C3 levels and severity of NAFLD. Individuals with ultrasonographic hepatosteatosis combined with abnormal liver enzymes are considered to be a more severe form of NAFLD^8^,^18^. Therefore, we divided NAFLD patients into two groups according to whether their serum ALT levels were elevated (≥50 U/L) or not. We found that the medium (interquartile range) of serum complement C3 levels was 1.25 (1.16–1.36) g/L in NAFLD patients with normal range of serum ALT, but the levels increased to 1.33 (1.23–1.46) g/L in NAFLD patients with elevated serum ALT (Mann-Whitney U test, *P* < 0.001). We also found that the prevalence of NAFLD with elevated serum ALT, a more severe form of NAFLD, was positively correlated with serum complement C3 levels. The prevalence was 0.43%, 1.53%, 4.62%, and 10.63% in participants with serum complement C3 in quartiles 1, 2, 3, and 4, respectively (*P* for trend <0.001). These findings suggest that serum complement C3 levels were not only associated with NAFLD prevalence, but also associated with disease severity.

### The association between serum complement C3 and NAFLD is independent of obesity and metabolic syndrome

Obesity and metabolic syndrome are two major factors that closely associate with NAFLD, and these two factors may act as cofactors for the link between serum complement C3 levels and NAFLD. To explore whether the link between serum complement C3 levels and NAFLD is independent of obesity and metabolic syndrome, we excluded 2383 participants who were obese (BMI ≥ 25 kg/m^2^) and/or had metabolic syndrome for subgroup analysis. Of the remaining 5157 lean and metabolic syndrome-free participants, 693 (13.43%) met diagnostic criteria of NAFLD. Serum complement C3 levels remained to be significantly higher in NAFLD patients compared with those without (1.24 (1.15–1.33) g/L *vs.* 1.12 (1.04–1.22) g/L, *P *< 0.001). A positive correlation between serum complement C3 levels and prevalence of NAFLD was also observed among lean and metabolic syndrome-free participants. The prevalence was 3.55%, 10.54%, 16.97%, and 29.66%, in quartiles 1, 2, 3, and 4, respectively (*P* for trend <0.001). These results suggest that the link between serum complement C3 levels and NAFLD is not influenced by obesity and obesity-related metabolic disorders.

### Elevated serum complement C3 levels independently increases risk of NAFLD

We further performed a stepwise multiple regression analysis, to explore whether elevated serum complement C3 levels was independently associated with increased risk for NAFLD. We input 17 variables including age, gender, body mass index, waist circumference, systolic and diastolic blood pressure, ALT/AST, γ-glutamyltransferase, albumin, triglyceride, total cholesterol, HDL cholesterol, fasting blood sugar, serum uric acid, white blood cell, platelet count, and serum complement C3 into the original equation. We found that 16 variables remained in the final equation and predicted to be closely associated with risk for NAFLD (Table 3). A noticeable finding was that serum complement C3 was significantly associated with risk for NAFLD (OR = 5.231; 95% CI: 3.169–8.635). This finding provides strong evidence that elevated serum complement C3 is a significant independent factor associated with risk of NAFLD.

## Discussion

This cross-sectional study revealed that NAFLD patients had higher serum complement C3 levels, which in further analysis turned out to be positively associated with both NAFLD prevalence and severity. This relationship was independent of age, gender, body mass index, waist circumference, systolic and diastolic blood pressure, ALT/AST, γ-glutamyltransferase, uric acid and other metabolic syndrome features.

The role of complement system in metabolic disorders has been extensively investigated during recent years^19^. Serum complement C3 levels are observed to be independently associated with dyslipidemia^20^, coronary heart disease^21^,^22^,^23^, diabetes^24^, and hypertension^25^. In this study, we provided evidences for the first time that serum complement C3 level is independently associated with NAFLD in non-obese and metabolic syndrome free population, and the C3 levels are positively associated with severity of NAFLD. Our results suggested a significant role of complement C3 in NAFLD.

The underlying mechanism by which complement C3 interacts with NAFLD remained unknown, but several hypotheses were proposed. Firstly, complement components, particularly C3, play important role in lipid metabolism^20^. C3 is mainly synthesized in hepatocytes but can also be secreted by adipose tissue and recently discovered to be present in lipoprotein particles, such as high density lipoprotein and chylomicron^26^,^27^. C3 knockout mice had 58% higher serum triglyceride levels and altered lipoprotein profile with more low-density lipoprotein cholesterol and very-low-density lipoprotein triglycerides, indicating a potential role of C3 in lipid metabolism regulation^28^. Secondly, innate immune response is a part of the most widely accepted theory of NAFLD pathogenesis, “two hit model”. The first hit is correlated with insulin resistant with fat accumulation and then on this basis, oxidative stress induces varied inflammatory cytokines as well as great number of adipokines, inducing the second hit^29^. Complement system, as a major player in innate immune response, might be activated by the first hit and forming the second hit in NAFLD pathogenesis^30^. The third, hepatocyte apoptosis, activated by intracellular stress of membrane-bound organelles and molecule cascade including caspases 3 and 7, or cleavage of cytokeratin 18, is a key morphologic and pathogenic feature of human NAFLD and NASH^31^. Complement system was found to be activated by apoptosis and can help with recognition and clearance of apoptotic cells^32^. Thus elevation of serum complement C3 might act as a protective response in NAFLD mediated by apoptosis. Last but not least, acylation-stimulating protein (ASP), a C3 derivative involved in adipocyte lipid metabolism by stimulating triglyceride synthesis, was found increased in NAFLD patients^33^. On one hand, this was considered as a result from “ASP resistance” of adipose tissue in NAFLD patients, but on the other hand, hepatic C3 activation can also be the trigger^12^. ASP can promote triglyceride accumulation in liver cells; thereby forming a vicious cycle that complement activation exacerbates steatosis, and in return steatosis promotes complement activation.

Our study has some limitations. Firstly, although ultrasound-based diagnosis of NAFLD is widely accepted under clinical circumstances as a noninvasive cost-effective screening for NAFLD with sensitivity of 89% and specificity of 93%, it cannot replace pathological study as gold standard in disease diagnosis^34^,^35^. Further explore the relationship between serum C3 levels and histological severity of NAFLD would be of great interest. Secondly, with the nature of cross-sectional study, no causal relationship can be identified. Further prospective studies are needed in this concern. Nevertheless, current analysis revealed that serum complement C3 is positively associated with prevalence of NAFLD and disease severity as well.

Our study has significant importance for NAFLD therapy and prevention. C3, as a key in innate immune system and potential regulator of lipid metabolism as mentioned above, may contribute significantly to pathogenesis of NAFLD. Thus, developing medication targeting C3/C3 receptor or intervening C3 activation might be effective for NAFLD treatment. Moreover, high serum C3 levels can also serve as an independent predictor for NAFLD. With well-established relationship between C3 and NAFLD prevalence as well as disease severity in addition to the role of potential therapeutic target, prognostic role of C3 is also worth investigating.

In conclusion, current cross-sectional study in a large Chinese population revealed that serum complement C3 level is positively associated with prevalence and severity of NAFLD, and this association is independent of obesity and metabolic syndrome.

## Additional Information

**How to cite this article**: Xu, C. *et al.* Serum complement C3 levels are associated with nonalcoholic fatty liver disease independently of metabolic features in Chinese population. *Sci. Rep.* **6**, 23279; doi: 10.1038/srep23279 (2016).

## Figures and Tables

**Table 1:** Characteristics of study participants with or without NAFLD.

Variables	With NAFLD	Without NAFLD	*t* value	*P* value
*n* (male/female)	2070 (1623/447)	5470 (3446/2024)	161.810^a^	<0.001
Age (year)	51.0 (14.2)	48.4 (15.6)	6.376	<0.001
Body mass index (kg/m^2^)	25.67 (2.70)	22.46 (2.43)	49.770	<0.001
Waist circumference (cm)	88.7 (8.1)	80.5 (8.0)	39.422	<0.001
Systolic blood pressure (mmHg)	129.7 (14.9)	122.3 (16.0)	18.269	<0.001
Diastolic blood pressure (mmHg)	80.0 (9.6)	75.1 (9.5)	19.768	<0.001
Alanine aminotransferase (U/L)	26.0 (19.0–41.0)	16.0 (12.0–22.0)	34.604^b^	<0.001
Aspartate aminotransferase (U/L)	24.0 (20.0–30.0)	20.0 (17.0–24.0)	21.571^b^	<0.001
γ-Glutamyltransferase (U/L)	32.0 (23.0–49.0)	19.0 (15.0–27.0)	34.085^b^	<0.001
Triglyceride (mmol/L)	1.60 (1.18–2.22)	0.97 (0.71–1.34)	36.384^b^	<0.001
Total bilirubin (μmol/L)	14.8 (6.2)	14.6 (6.6)	1.729	0.084
Albumin (g/L)	47.1 (2.6)	46.6 (2.7)	6.699	<0.001
Total cholesterol (mmol/L)	5.03 (4.44–5.71)	4.75 (4.15–5.40)	11.976^b^	<0.001
HDL cholesterol (mmol/L)	1.42 (1.25–1.61)	1.62 (1.43–1.88)	25.196^b^	<0.001
LDL cholesterol (mmol/L)	2.81 (0.76)	2.60 (0.77)	10.312	<0.001
Fasting plasma glucose (mmol/L)	5.10 (4.73–5.60)	4.90 (4.60–5.27)	13.943^b^	<0.001
Serum uric acid (μmol/L)	376.5 (80.5)	317.9 (77.0)	29.126	<0.001
White blood cell (×10^9^/L)	6.4 (5.5–7.5)	5.7 (4.9–6.7)	19.846^b^	<0.001
Platelet count (×10^9^/L)	215.0 (184.0–250.0)	209.0 (178.0–242.0)	5.085^b^	<0.001
Serum complement C3 (g/L)	1.26 (1.17–1.37)	1.13 (1.04–1.23)	31.888^b^	<0.001

Data are expressed as mean (SD) or median (IQR).

^a^*χ*^2^ value;

^b^*Z* value; HDL, high-density lipoprotein; LDL, low-density lipoprotein.

**Table 2:** Association of serum complement C3 levels with prevalence rate of NAFLD.

ComplementC3 quartiles	Total	NAFLD	PR%	PR	χ^2^	*P*
Quartile 1	1459	145	7.78	1.00		
Quartile 2	1474	368	19.45	2.50		
Quartile 3	1502	553	31.56	4.06		
Quartile 4	1481	1004	49.41	6.35	1170.393	<0.001

PR%, prevalence rate; PR, prevalence ratio.

**Table 3:** Risk factors associated with the presence of NAFLD.

Variables	β	SE	Wald χ^2^	*P*	OR (95% CI)
Age (year)	0.028	0.003	79.970	<0.001	1.028 (1.022–1.035)
Male gender	0.549	0.106	26.884	<0.001	1.731 (1.407–2.129)
Body mass index (kg/m^2^)	0.284	0.018	248.144	<0.001	1.328 (1.282–1.376)
Waist circumference (cm)	0.039	0.006	41.316	<0.001	1.040 (1.028–1.053)
Diastolic blood pressure (mmHg)	0.025	0.005	28.278	<0.001	1.025 (1.016–1.034)
ALT/AST ratio	1.649	0.107	235.661	<0.001	5.201 (4.213–6.419)
γ-Glutamyltransferase (U/L)	0.005	0.001	18.993	<0.001	1.005 (1.003–1.008)
Albumin (g/L)	0.081	0.015	30.619	<0.001	1.084 (1.054–1.116)
Triglyceride (mmol/L)	0.214	0.055	15.234	<0.001	1.238 (1.112–1.379)
Total cholesterol (mmol/L)	0.156	0.049	9.944	0.002	1.169 (1.061–1.288)
HDL cholesterol (mmol/L)	−1.352	0.171	62.530	<0.001	0.259 (0.185–0.362)
Fasting plasma glucose (mmol/L)	0.123	0.034	12.925	<0.001	1.131 (1.058–1.209)
Serum uric acid (μmol/L)	0.003	0.001	44.775	<0.001	1.003 (1.002–1.004)
White blood cell (×10^9^/L)	0.064	0.024	6.923	0.009	1.066 (1.016–1.118)
Platelet count (×10^9^/L)	0.003	0.001	20.754	<0.001	1.003 (1.002–1.005)
Serum complement C3 (g/L)	1.655	0.256	41.869	<0.001	5.231 (3.169–8.635)

β, partial regression coefficient; SE, standard error of partial regression coefficient; OR, odds ratio; CI, confidence interval; HDL, high-density lipoprotein.
